# Effects of an irregular bedtime schedule on sleep quality, daytime sleepiness, and fatigue among university students in Taiwan

**DOI:** 10.1186/1471-2458-9-248

**Published:** 2009-07-19

**Authors:** Jiunn-Horng Kang, Shih-Ching Chen

**Affiliations:** 1 Sleep Science Center, Taipei Medical University Hospital, No. 252 Wu-Xing Street, Taipei 110, Taiwan, Republic of China; 2 Department of Physical Medicine and Rehabilitation, Taipei Medical University Hospital, No. 252 Wu-Xing Street, Taipei 110, Taiwan, Republic of China

## Abstract

**Background:**

An irregular bedtime schedule is a prevalent problem in young adults, and could be a factor detrimentally affecting sleep quality. The goal of the present study was to explore the association between an irregular bedtime schedule and sleep quality, daytime sleepiness, and fatigue among undergraduate students in Taiwan.

**Methods:**

A total of 160 students underwent a semi-structured interview and completed a survey comprising 4 parts: Pittsburgh Sleep Quality Index (PSQI), Epworth Sleepiness Scale (ESS), Fatigue Severity Scale (FSS), and a rating of irregular bedtime frequency. Participants were grouped into 3 groups in terms of irregular bedtime frequency: low, intermediate, or high according to their 2-week sleep log. To screen for psychological disorders or distress that may have affected responses on the sleep assessment measures, the Chinese health questionnaire-12 (CHQ-12) was also administered.

**Results:**

We found an increase in bedtime schedule irregularity to be significantly associated with a decrease in average sleep time per day (Spearman r = -0.22, p = 0.05). Multivariate regression analysis revealed that irregular bedtime frequency and average sleep time per day were correlated with PSQI scores, but not with ESS or FSS scores. A significant positive correlation between irregular bedtime frequency and PSQI scores was evident in the intermediate (partial r = 0.18, p = 0.02) and high (partial r = 0.15, p = 0.05) frequency groups as compared to low frequency group.

**Conclusion:**

The results of our study suggest a high prevalence of both an irregular bedtime schedule and insufficient sleep among university students in Taiwan. Students with an irregular bedtime schedule may experience poor sleep quality. We suggest further research that explores the mechanisms involved in an irregular bedtime schedule and the effectiveness of interventions for improving this condition.

## Background

Some behaviours or activities are detrimental to normal sleep have been suggested. These "inadequate sleep hygiene" behaviours include irregular sleep schedules, frequent or prolonged daytime naps, excessive alcohol consumption before bedtime, and staying on one's bed for non-sleep-related activities [1-3]. Accordingly, adequate sleep hygiene is considered to be an important adjuvant for treating patients with insomnia or other sleep disturbances [1,3,4]. However, in the case of normal subjects, who are unaffected by these pathological conditions, the association between sleep hygiene and sleep itself is surprisingly inconsistent [5-8].

Sleep is regulated by two main processes: the sleep homeostatic drive, influenced by experienced durations of sleep and wakefulness, and the circadian system, an intrinsic pacemaker involving a pathway from the suprachiasmatic nucleus to the hypothalamus [9]. The circadian system has complex interactions with daily behaviours, known as entraining factors. It is thought that having a regular bedtime schedule can strengthen the circadian rhythm; and is beneficial for achieving a good quality of sleep [10]. Even one-night alteration to a sleep schedule can be sufficient to induce difficulties with sleep initiation and maintenance. Taub et al. reported that an acute shift in sleep schedules of 2 hours without altering total duration of sleep can decrease cognitive and psychological functioning in a laboratory setting [11-14]. However, it is unclear if this finding can be generalised to real life conditions over long durations.

Another condition associated with frequent changes in the sleep-wake schedule is in the case of shift workers. Shift work is found to adversely affect sleep and general health, including decreased sleep quality, altered sympathetic activity, increased risk of cardiovascular events, and reduced cognitive performance [15-17]. However, the changing sleep/wake pattern of shift workers differs greatly from that of young adults; the phase shifts of the latter group are shorter but more irregular than the former. This suggests that the data obtained from shift workers may not adequately represent those for young adults with an irregular sleep schedule.

Although the prevalence varies, many adolescents and young adults are reported to have an irregular sleep schedule and a tendency to have a delayed sleep phase [18-22]. Furthermore, a remarkable degree of problems associated with sleeping and poor sleep quality have been observed in university students of many Western countries [18-22]. However, to our knowledge, the data regarding sleep patterns and habits in the Chinese is limited. It is necessary to investigate this issue within the Chinese population because sleep habits are affected by ethnicity, social factors, and culture [20,22]. Our aim was to investigate sleep quality and associated daytime effects in Chinese undergraduate students. We were particularly interested in determining how bedtime schedule relates to sleep quality and daytime functioning.

## Methods

### Sampling and study population

The Taipei Medical University Hospital Review Board approved the present study, and all participants provided informed consent. The participants were selected randomly from first-year undergraduate students from a medical university in Taipei, Taiwan. We excluded subjects with a history of either chronic medical or psychotic disorders, as well as those currently on medication. Of 197 students interviewed, 160 (81.2%) completed the survey; 81 males and 79 females, with a mean age of 20.3 ± 1.9 years. were included in the study.

### Measures and data management

**Figure 1:**
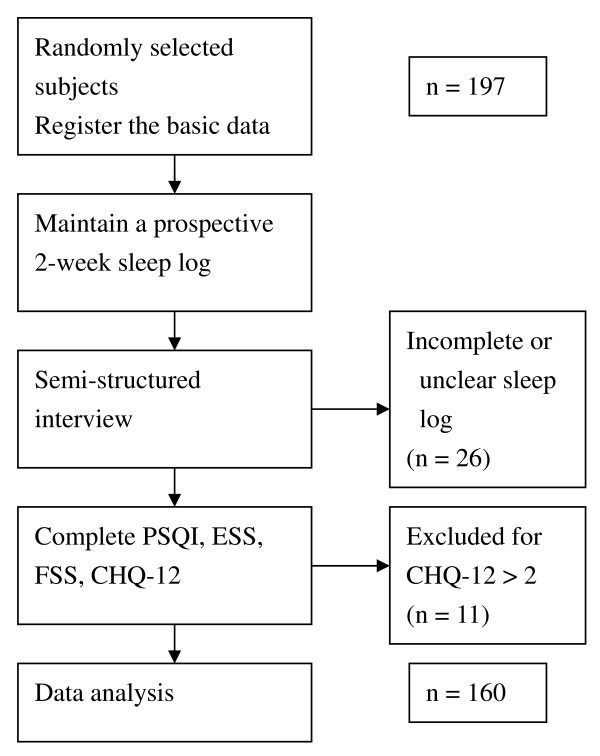
**Flowchart of the survey and data collection process**.

A flowchart of the survey and data collection process is presented in Figure 1. Participants were first asked to maintain a prospective 2-week sleep log. Subsequently, they underwent a semi-structured interview, and the quality of entries in their sleep log was reviewed at that time. Sleep schedule and average daily sleep time data were obtained and analyzed from the sleep logs. To decrease the difficulty and uncertainty of scoring, irregular bedtime frequency was defined as the number of nights when a greater than 1 hour shift in bedtime from the usual bedtime had occurred in the past 2 weeks. The subjects were assigned to 1 of 3 irregular bedtime frequency groups: low, ≤ 1 night per week; intermediate, 1–3 nights per week; and high, ≥ 3 nights per week or not having a regular bedtime.

The interview involved the completion of 3 questionnaires evaluating sleep quality, daytime sleepiness, and fatigue. Sleep quality was assessed using the Pittsburgh Sleep Quality Index (PSQI), which is widely accepted as both valid and reliable [23,24]. The PSQI includes 19 items, and yields a score from 0 (good quality) to 21 (poor quality). Sleep onset latency and sleep efficiency, defined as the actual sleep time divided by the time in bed, are also obtained with the PSQI. Daytime sleepiness was assessed using the Epworth Sleepiness Scale (ESS), a widely used and reliable predictor of daytime sleepiness [25]. The ESS employs a 4-point scale to rank the chances of a subject falling asleep in different scenarios. Fatigue was evaluated using the Fatigue Severity Scale (FSS); this scale includes 9 questions, yields a total score from 9 (no fatigue) to 63 (severe fatigue), and has been previously applied in clinical evaluations of fatigue [26,27].

Subjects were also administered the CHQ-12 (Chinese health questionnaire-12) in order to screen for psychological disorders or distresses that may have affected responses on the sleep assessment measures. The CHQ-12 includes 12 items, and has been shown as valid for use with the Chinese population [28]. We excluded 11 students from the study for having a CHQ-12 score greater than 2 points.

### Statistical analysis

There were five major dependent variables: sleep onset latency, sleep efficiency, and scores for each of the PSQI, ESS, and FSS. Student's *t*-test was used to compare males and females with respect to each variable. Chi-square test and Spearman's correlation were used to analyse relationships between irregular bedtime frequency and average sleep time per day.

To control for the potential confounding effects of sleep insufficiency, multivariate linear regression adjusted for average daily sleep time was used to analyse how irregular bedtime frequency related to scores for each of the PSQI, ESS, and FSS. The analysis was performed by setting the intermediate and high FIB groups as dummy variables in comparison to the low FIB group. The significance level was set as p < 0.05.

## Results

There was no statistical difference between males and females for any variable; therefore, the data for both genders was combined in all subsequent analyses. Across all subjects, the mean sleep onset latency was 14.2 ± 10.6 min, and the mean average daily sleep time was 6.7 ± 1.3 h. Mean scores for the FSS, ESS, and PSQI were 38.2 ± 8.9, 6.3 ± 3.3, and 4.9 ± 2.4, respectively. The data for all variables and subjects are summarised in Table 1. Poor sleep quality (PSQI score >5), daytime sleepiness (ESS score >10), and fatigue (FSS score ≥ 36) were each reported by a considerable proportion of participants, 33.8%, 14.4%, and 37.5%, respectively.

**Table 1:** Mean (standard deviation (SD)) results of all measures for both genders and all subjects

**Variable**	**Female (n = 81)**	**Male (n = 79)**	**Total (n = 160)**
**SOL (min)**	15.2 (11.3)	12.9 (9.8)	14.2 (10.6)
**SE (%)**	89.0 (14.4)	91.1 (10.5)	90.1 (12.6)
**ADST (h)**	6.8 (1.4)	6.6 (1.1)	6.7 (1.3)
**ESS score**	6.7 (3.2)	5.9 (3.4)	6.3 (3.3)
**FSS score**	38.0 (8.5)	38.5 (9.4)	38.2 (8.9)
**PSQI score**	5.0 (2.4)	4.8 (2.3)	4.9 (2.4)

SOL, sleep onset latency; SE, sleep efficiency; ADST, average daily sleep time; ESS, Epworth Sleepiness Scale; FSS, Fatigue Severity Scale; PSQI, Pittsburgh Sleep Quality Index

There was no significant difference (p < 0.05) between genders for any variable

With respect to irregular bedtime frequency, 26.9% of the participants were in the low frequency group (<1 night per week), 38.8% were in the intermediate frequency group (1–3 nights per week), and 34.4% were in the high frequency group (>3 nights per week). Nearly half of all subjects (46.9%) reported an average daily sleep time that was less than 7 h. It is worth noting that average daily sleep time was negatively correlated with irregular bedtime frequency (r = -0.22, p < 0.05). The distribution of average daily sleep time across irregular bedtime frequency groups is shown in Table 2.

**Table 2:** Number (%) of subjects in each irregular bedtime frequency group with an average daily sleep time <7 h/day, 7–8 h/day, or >8 h/day

ADST*	**Irregular bedtime frequency^+^**			**Total**
		
	**Low**	**Intermediate**	**High**	
**<7 h/day**	14 (8.8)	26 (16.3)	35 (21.9)	75 (46.9)
**7–8 h/day**	17 (10.6)	24 (15.0)	10 (6.3)	51 (31.9)
**>8 h/day**	12 (7.5)	12 (7.5)	10 (6.3)	34 (21.3)

**Total**	43 (26.9)	62 (38.8)	55 (34.4)	160 (100)

ADST, average daily sleep time

^+ ^An irregular bedtime event was defined as a shift from the usual bedtime of >1 h. This occurred <1 night/week for the low-frequency group, 1–3 nights/week for the intermediate-frequency group, and >3 nights/week for the high-frequency group.

*Chi-Square analysis indicated that average daily sleep time differed significantly across the irregular bedtime frequency groups (χ^2 ^= 11.68, p = 0.022); as supported by a significant Spearman correlation (r = -0.22, p = 0.05).

Multivariate regression analysis revealed irregular bedtime frequency and average daily sleep time had a significant positive correlation with PSQI scores (r = 0.61, p < 0.001), but not with ESS or FSS scores. With adjustment for average daily sleep time, this positive correlation of irregular bedtime frequency with PSQI scores was more evident in the intermediate (partial r = 0.18, p = 0.02) and high (partial r = 0.15; p = 0.05) frequency groups than the low frequency group (Table 3).

**Table 3:** Results of multivariate regression analysis of irregular bedtime frequency and average daily sleep time on PSQI, ESS, and FSS scores

	**ESS**	**FSS**	**PSQI**
**Intermediate FIB (p-value)**	(0.389)	(0.132)	(0.02*)
Zero-order r	-0.05	0.05	0.05
Partial r	0.07	0.12	0.18
**High FIB (p-value)**	(0.015*)	(0.124)	(0.05*)
Zero-order r	0.18	0.09	0.20
Partial r	0.19	0.12	0.15
**ADST (p-value)**	(0.314)	(0.359)	(<0.001*)
Zero-order r	0.02	-0.11	-0.59
Partial r	0.08	-0.07	0.56

**Total r (p-value)**	0.20	0.17	0.61
	(0.09)	(0.196)	(<0.001*)

FIB, frequency of irregular bedtime; ADST, average daily sleep time; ESS, Epworth Sleepiness Scale; FSS, Fatigue Severity Scale; PSQI, Pittsburgh Sleep Quality Index; r, correlation coefficients.

The multivariate regression analysis was performed by setting the intermediate and high FIB groups as dummy variables in comparison to the low FIB group

*p < 0.05

## Discussion

Our findings indicated that students with a frequently irregular bedtime had poor sleep quality, even after adjusting for the sleep time. We also found negative trends between an irregular bedtime schedule and daytime functioning; although those concerning ESS and FSS scores were not statistically significant. The consequences of an irregular bedtime schedule may be less significant than those of sleep insufficiency. However, the adverse effects of an irregular bedtime schedule on sleep should not be overlooked. We postulate that an irregular bedtime schedule affects sleep quality by disturbing the circadian rhythm. Previous studies have shown that disturbances of the circadian rhythm produce alterations in sleep architecture and sleep quality known to be associated with fatigue, vigilance problems, decreased productivity, and negative health effects [10,29].

We found that students with a frequently irregular bedtime also had a relatively short average sleep time per day, which could put them at risk of sleep insufficiency. This finding may be partially explained by the daytime activity schedules (for classes or recreation) of these students limiting their ability to get enough sleep in the morning. In addition, environmental factors like light or noise can also be detrimental to people trying to sleep late into the morning or day. Many studies have reported that an accumulated sleep deprivation induces impairments of cognition, vigilance, and memory, and disturbances of mood [30-33]. We suggest that students with an irregular bedtime schedule should be encouraged to maintain a regular bedtime, and to increase their total sleep time. These changes might help to increase the sleep quality and daytime functioning of these students.

The mechanisms by which an irregular sleep schedule develops in young adults are not well understood; however, they are likely to be multi-factorial, including biological, behavioural, and social factors. Two endogenous circadian rhythm patterns of alertness and sleepiness have been observed in the general population, in accordance with which people may be classified as being of a morning type (M-type) or an evening type (E-type) [34]. The sleep/wake schedule of these types differs, with the E-type associated with irregular sleeping habits and an increased need for sleep [35,36]. In addition to biological factors, social cues, activity types and levels, environmental factors, and motivation to maintain a regular bedtime all also play important roles in entraining the sleep-wake cycle by adjusting the intrinsic (endogenously regulated) circadian rhythm [10]. Further research is needed to clarify the underlying biological and behavioural mechanisms by which an irregular bedtime schedule develops.

Our results indicate that there is a high prevalence of both an irregular bedtime schedule and short average daily sleep time among undergraduate students in Taiwan. These findings are compatible with those of studies from other countries, thereby suggesting that poor sleep in university students may be a universal and prevalent problem of modern society [18,19,21]. In reviewing the research on sleep patterns in university students, Hicks et al. found the median sleep time to have dropped by about 1 hour over the past 20 years [37]. Lifestyle, social and academic schedules, and insufficient sleep education could all contribute to the aetiology of chronic sleep insufficiency and poor sleep in university students [18,19,38]. Some approaches to enhancing the sleep hygiene and relevant knowledge of university students have been developed, but the effectiveness of these remains to be confirmed [38].

The present study has some limitations. First, the self-reported estimation of average daily sleep time and irregular bedtime frequency may have been influenced by subjects' cooperation and a recall bias. An objective assessment method such as actigraphy is required for more accurate measurements of these parameters. Moreover, the university medical students we studied might not be representative of the more general population of university students in Taiwan. A randomised, population-based study with a large sample size will be required to confirm our findings. A further limitation of our cross-sectional study is the difficultly in establishing causality. It is possible that subjects who have poor sleep quality (e.g., sleep onset difficulty or sleep fragmentation) could subsequently develop inadequate sleep habits, including an irregular sleep schedule. Longitudinal studies are thus required to clarify the cause-and-effect relationships between an irregular bedtime schedule and poor sleep quality. Finally, there are many different inadequate sleep habits and sleep-related behaviours likely to have a detrimental impact on sleep quality in normal subjects. An irregular bedtime schedule was the only one of these factors investigated in the present study, and future studies should take more of these factors into consideration.

## Conclusion

Our results suggest that there is a high prevalence of both an irregular bedtime schedule and insufficient sleep among university students in Taiwan. Students with an irregular bedtime schedule may also experience poor sleep quality. Further research exploring the mechanisms involved in an irregular bedtime schedule and the effectiveness of interventions for improving this condition should be conducted.

## Competing interests

The authors declare that they have no competing interests.

## Authors' contributions

JHK participated in designing the study, conducting the interviews, performing data analysis, and drafting the manuscript. CSC participated in designing and coordinating the study. Both authors read and approved the final manuscript.

## Pre-publication history

The pre-publication history for this paper can be accessed here:


